# Production of Chitosan/Hyaluronan Complex Nanofibers. Characterization and Physical Properties as a Function of the Composition

**DOI:** 10.3390/polym12092004

**Published:** 2020-09-03

**Authors:** Christian Enrique Garcia Garcia, Félix Armando Soltero Martínez, Frédéric Bossard, Marguerite Rinaudo

**Affiliations:** 1 Departamento de Ingeniería Química-CUCEI. Blvd. M. García Barragán #1451, C.P., Universidad de Guadalajara, Guadalajara, Jalisco 44430, Mexico; jfasm@hotmail.com; 2 Université Grenoble Alpes, CNRS, Grenoble INP, LRP, Institute of Engineering University of Grenoble Alpes, 38000 Grenoble, France; frederic.bossard@univ-grenoble-alpes.fr; 3 Biomaterials Applications, 6 Rue Lesdiguières, 38000 Grenoble, France

**Keywords:** chitosan, hyaluronan, polyelectrolyte complex, crosslink, H-bonds, amide bonds, electrospinning, nanofibers, formic acid

## Abstract

In this work, optimized conditions for preparation of chitosan and hyaluronan polyelectrolyte complex are proposed. The objective was to produce new biomaterials being biocompatible and bioresorbable in the body as well as approaching the extracellular matrix (ECM) structure. These materials will be tested for chondrocyte development in tissue engineering and wound healing applications. Nanofibers made of the polyelectrolyte complex (PEC) were successfully manufactured by electrospinning, and casted films were used as a model for properties comparison. To our knowledge, it is the first time that stable chitosan/hyaluronan fibers are produced, which were observed to be long-lasting in buffer at pH~7.4. The role of thermal treatment at 120 °C for 4 h is examined to control the degree of swelling by crosslinking of the two polysaccharides by H-bonds and amide bonds formation. The properties of the materials are tested for different PEC compositions at different pH values, based on swelling and solubility degrees, diameters of nanofibers and mechanical performances. The influence of the solvent (acidic potential and composition) utilized to process biomaterials is also examined. Acid formic/water 50/50 *v/v* is observed to be the more appropriated solvent for the carried-out procedures.

## 1. Introduction

The nanofibrous structure closely mimics the structure of the native extracellular matrix (ECM) in which cells normally reside in the body [1,2]. To ensure the growth of cells, these supports must be porous, should have good water-holding capacity, and should allow easy permeation of gases and metabolites. Nanofibers due to their unique properties like large surface area, high porosity, and increased mechanical strength are considered as ideal material for scaffold preparation. It has also been found that nanofibers help in promoting adherence, growth, and proliferation of seeded cells and successful development of tissue-engineered constructs. In addition, electrospinning is a cost-effective, simple, and versatile method which can be used for fabrication of a variety of nanofibers at a large scale [3].

Electrospinning of soluble polymers at high voltage is a simple technique to produce micro- to nanoscale nanofibers. The large area to volume ratio and high porosity of electrospun fibers promotes the adhesion of cells and cell growth [2]. This technique was previously used in our laboratory to produce pure and stable chitosan fibers [4,5]. The main electrospinning parameters were studied on chitosan/polyethylene oxide (PEO) blends in acetic acid at 50% [6].

Chitosan (CS) is a pseudo natural polysaccharide with important properties for biomedical applications: it is biocompatible, biodegradable in the body, available on large scale from crustaceous organisms. It remains insoluble over pH~7 but solubilized in acidic conditions and becomes a positively charged polymer. Due to those characteristics, it is selected by many authors as a basis for polyelectrolyte complexes when mixed with polyanionic polymers [7].

Hyaluronan (HA), also called hyaluronic acid, is a natural polysaccharide and a critical component of natural ECM being widely used in tissue engineering and regenerative medicine [2,8,9,10,11]. These authors produced HA fibers using dimethylformamide (DMF)/water mixed solvents in different volume ratios (0:1, 0.25:1, 0.5:1, and 1:1, respectively) [8]. Nanofibers have been produced by electrospinning of HA using a mixture of 25% aqueous ammonium solution and N-methylpyrrolidone in ratio 2:1 [9]. Fibers were also obtained using NH_4_OH (25% in water) and DMF in a ratio 2:1 [10]. Due to difficulties to produce HA fibers, it was proposed to electrospin the blend HA/PEO giving a core–shell structure [11]. In these conditions, HA fibers are soluble in aqueous medium. The HA nanofibrous membranes electrospun from HA solution in the 1:1 ratio of DMF/water mixed solvent were stabilized by immersing in concentrated hydrochloric acid solution for 10 min [8]. The membrane was stable in the deionized water in a gel state for a short time after being stabilized in HCl solution for ten minutes but these nanofibers were quite stable in acid solution for several weeks. This behavior is related to formation of a physical gel at pH~2.5 formed in HA solution [12]. A thiolated HA derivative, 3,3′-dithiobis(propanoic dihydrazide)-modified HA (HA-DTPH) was electrospun in presence of PEO. It was found that the electrospun HA-DTPH scaffolds swelled to form hydrogels even without adding crosslinker, indicating that a spontaneous cross-linking reaction happened from air-induced oxidation of thiols to disulfide linkages during electrospinning [13]. These results concerning electrospinning of pure HA was obtained in many solvent conditions but the stability of the fibers in aqueous medium remains low due to the high solubility of HA.

PEO is a biocompatible, biodegradable, non-immunogenic, and non-toxic synthetic polymer [2]. It does not interact with proteins and most other biological molecules, and supports the development of new tissue. Furthermore, the water holding capacity of PEO nanofibers stimulates wound healing [2]. PEO is often mixed with other polymers since it increases the electrospinnability of the final blend and, as a water-soluble polymer, it can be easily extracted from the new fibers produced to get polymeric materials stable in aqueous medium.

To obtain stable nanofibers in aqueous medium, PEC was introduced. PECs are a type of composite fiber produced by mixing two polymers with opposite charges stabilized by strong electrostatic interactions between the polycation and polyanion. [7,14,15,16,17,18]. The materials based on PECs are often characterized by a degree of swelling depending on pH and external ionic concentration [19]. Often, chitosan is selected as polycation due to its specific properties and associated with synthetic or natural polyanions.

Interesting nanofibers were prepared from chitosan/alginate polyelectrolyte complex [1]. Chitosan/PEO and alginate/PEO solutions have been mixed for electrospinning with different volume ratios (20/80, 30/70, 40/60, 50/50, and 80/20). Those nanofibrous scaffolds were able to promote the adhesion and proliferation of cells. This result indicated that nanofibers made of a polyelectrolyte complex may be stable in aqueous medium due to strong electrostatic interactions. The degree of swelling after extraction of PEO in water decreases when yield in chitosan increases. Fibers have also been produced by coaxial electrospinning; the alginate solution blended with PEO was used to form the inside (core) of the nanofibers and the chitosan solution mixed with PEO composed the outside [20].

Due to difficulties to electrospin pure HA, Ma and co-workers successfully electrospun HA solubilized in water (W)/formic acid (FA) (25/75 *w/w*) with positively charged chitosan in W/FA (20/80 *w/w*) [14] or by mixing HA solution in W/FA/DMF (5.0/2.5/2.5, *w/w*) and chitosan solution in W/FA(1/9, *w/w*) at the different blending weight ratio from 9/1 to 5/5 [14]. A bilayer chitosan/hyaluronan CS–HA-PEO material was produced by sequential electrospinning of HA-PEO onto a freshly formed CS-PEO layer; the ratio of the thickness of the CS:HA layers was 2:1 [21]. It is shown that the mechanical properties (strength and stiffness) increase. In addition, it was demonstrated a significantly higher number of living cells on the surface of the CS–HA compared with CS with a better biocompatibility. Chitosan/hyaluronan hybrid biomaterials proposed in cartilage tissue engineering were formed by wet-spinning of chitosan solubilized in 2% acetic acid immersed in presence of calcium solution and coated with hyaluronan [22,23,24,25]. The same technique was proposed to establish medical use for ligament and tendon tissue engineering [26].

It has been shown that the CS/HA hybrid support serves as an ideal biomaterial to create a three-dimensional (3D) scaffold with adequate strength, high cellular adhesivity, and excellent support for chondrogenesis, preserving the phenotype and enhancing production of type II collagen (with increase of type II/ type I collagen ratio) [22]. Data obtained on CS/HA hybrid fibers indicate that materials including HA provide excellent adhesivity for seeded chondrocytes and enhance their biological behavior on the 3D scaffolds with different pore sizes [25]. In addition, it is shown that large pores (400 μm) in the structured 3D materials have much better mechanical properties and better cartilage regeneration [24,25].

In the present work, after production of chitosan and hyaluronan fibers by electrospinning, PEC made of chitosan and hyaluronan were prepared from chitosan and hyaluronan solutions in the same solvent at controlled –NH_2_/–COONa ratios. The stability of the materials obtained was studied on films casted from the PEC prepared in the same conditions as the nanofibers obtained by electrospinning. The role of thermal treatment on the complex stability is finally characterized by measuring the solubility and swelling degrees and mechanical properties.

## 2. Materials and Methods

Chitosan sample from Northern cold-water shrimp, *Pandalus borealis,* was utilized (Batch TM4778, code 42010, Primex Ehf, Siglufjordur, Iceland). Its weight-average molecular weight (*M*_W_) is 160 kg/mol and its degree of acetylation (DA), determined using ^1^H NMR, is 0.05. Hyaluronan sample was purchased from Soliance (Pomacle, France) with an average molecular weight, *M*_w_ = 540 kg/mol.

Formic acid (FA) (ACS reagent > 98%) from Sigma-Aldrich (lot #STBJ3705, Espoo, Finland) and Dulbecco’s Phosphate Buffered Saline (DPBS), pH = 7.4, (ref. 14190–094, Lot 2118924) from Gibco (Paisley, UK). Polyethylene oxide from Sigma-Aldrich has a molecular weight of 1 × 10^3^ kg/mol. Deionized water was used as solvent to prepare the solutions and all reagents and polymers were used as received without further purification.

### 2.1. Preparation of PEC Solutions

Chitosan and Hyaluronan homogeneous solutions were prepared separately at 4% (*w*/*w*) in water/formic acid mixtures W/FA at ratios 75/25, 50/50, and 25/75 (*v/v*) to obtain stable solutions. In these conditions, the total functional groups contents were 0.233 [–NH_2_]/L in chitosan and 0.1 [–COOH]/L in hyaluronan, respectively.

PEC systems are usually very difficult to process due to phase separation related to strong electrostatic interactions. The selection of a convenient solvent is essential hence W/AF mixtures were selected as previously proposed [14]. Subsequently, HA and CS solutions were mixed at different volume ratios corresponding to charge ratios *R*_c_ = 0.5, 1, 1.8, 2.35, and 3.0 under stirring getting a homogeneous blend.

### 2.2. Casting of PEC Films

A constant amount (~1.0 g) of each of the PEC mixtures was placed in a Teflon mold to obtain uniform polymer films with nearly the same thickness. The probes were stored at room temperature for 3 days until complete evaporation of the solvent until measuring constant dried weight.

Different samples of a regular shape were taken from the films obtained for future measurements of solubility, swelling degree in aqueous medium and mechanical properties in wet and dried states.

### 2.3. Electrospinning of PEC

In order to favor PEC spinnability, the addition of a 4% PEO *w/w* solution was needed. Using the same solvent as for the corresponding biopolymer mixture, final contents in PEC/PEO equal to 80/20 and 70/30 (*w/w*) were selected such as to preserve a high yield in polysaccharides in the fibers.

The prepared solutions were placed in a 5 mL plastic syringe fitted with a 21-gauge stainless steel needle. The syringe pump delivers solutions at specified flow rate vertically (model: KDS Legato 200, KD Scientific, Holliston, MA, USA), and electrospinning is realized with an applied voltage around 25 kV between the electrodes using a homemade dual high voltage power supplier (±20 kV, iseq GmbH, Radeberg, Germany). Then, the nanofibers were recovered on an aluminum film used as collector and fixed from 15 to 17 cm from the tip of the needle. The flow rates vary from 0.02 to 0.2 mL/h. The experiments were carried out at room temperature in closed Plexiglas^®^ box with relative humidity ranging between 40% and 60%. The produced nanofibers matrices were left in ambient conditions to evaporate excess of FA and water and reserved for further analyses.

### 2.4. Two Phases Fibers (Core-Shell Nanofibers)

In the case of fibers based on two polyelectrolytes, also named hybrid biomaterials, alginate fibers were coated with chitosan and chitosan fibers was coated with hyaluronic acid in the presence of calcium salt [22,25,26,27]. In this work, after modification of the technique proposed previously and few attempts, after electrospinning, PEO/CS nanofibers were immersed in HA solution (1 g/L) during 1 day and passed through CaCl_2_ saturated solution in EtOH/water (70/30 *v/v*) and washed in deionized water. The yield in hyaluronic acid is determined from the weight increase in the dried state. It comes that the mass ratio CS/HA = 1.38 corresponding to a charge ratio *R*_c_ = 3.44. These fibers were swollen in aqueous medium and found fully insoluble. Due to easier control of the polymer composition, homogeneous PEC biomaterials were preferred and no extensive work was performed on core–shell fibers.

### 2.5. Microscopy for Fibers Characterization

The scanning electron microscopy (SEM) analyses of the samples were performed at CERMAV, CNRS, Grenoble, France. The morphology of electrospun nanofiber samples was observed with a FEI QUANTA FEG 250 scanning electron microscope (ThermoFischer Scientific^TM^, FEI Co., Hillsboro, OR, USA) equipped with a field emission gun and operating at 2.5 kV. The nanofibers samples were coated with 3–4 nm gold/palladium prior to SEM imaging. The average fiber diameter (AFD) was calculated by a randomly selected diameter of 500 nanofibers from each sample.

### 2.6. Thermal Treatment of Films and Nanofibers

As proposed in literature, amide linkage is formed between –NH_2_ and –COOH under controlled thermal treatment at temperature between 80 and 120 °C up to 4 h [28,29,30,31,32]. In this work, after testing the kinetic of the reaction at 120 °C, CS and CS/HA complex under film and fiber mat morphologies were treated at 120 °C for 4 h in air conditions for structural stabilization.

### 2.7. NMR Characterization of Films and Nanofibers

Protons’ NMR spectra were recorded on a Bruker Avance III spectrometer (Bruker Corporation, Billerica, MA, USA) operating at a frequency of 400.13 MHz for ^1^H for which 5 mg samples were solubilized in 1 mL of D_2_O/DCl. Residual signal of the solvent was used as internal standard: HOD at 4.25 ppm at 353 K. Proton spectra were recorded with a 4006 Hz spectral width, 32,768 data points, 4.089 s acquisition times, 10 s relaxation delay, and 32 scans.

Analysis of the spectra allows the determination of nanofiber and film PEC composition, PEO and remaining solvent on selected samples. The yield of PEO remaining in the samples before and after PEO extraction in different conditions [4,33] is of special interest. The weight ratio of chitosan and hyaluronan are also obtained for each PEC studied when they are soluble in D_2_O.

### 2.8. Stability and Swelling Degrees

Stability of the new biomaterials was determined at equilibrium in phosphate buffer (DPBS) pH = 7.4 and expressed as final dried weight compared with the initial weight. Final dried weight (*W*_d_) compared with the initial dried weight (*W*_i_) allows to control the eventual partial solubility.

The swelling of the PEC films was also examined in terms of water loss between swollen state in PBS at pH = 7.4, in acidic (pH = 3) and basic medium (pH = 11), and final dried weight at room temperature. The wet swollen samples were weighed after blotting with tissue paper to remove excess surface water (*W*_w_). Accordingly, the dried samples were also weighted repeatedly until the mass became constant (*W*_d_). The measurements were carried out three times each. These values correspond to the first swelling. The average data were taken for the determination of swelling ratio S, expressed as mass (g) of retained water per gram of dried material, using the following equation:

(1) $$S\left({\mathrm{gH}}_{2}O/g\right)=\frac{W_{w}-W_{d}}{W_{d}}$$

where *W*_w_ (g) is the weight of the swollen nanofibrous mat or film and *W*_d_ (g) is the weight of the samples after drying at room temperature.

### 2.9. Mechanical Properties of Nanofiber Mat and Film

The measurements were carried out using ARES-G2 rheometer (TA Instruments, New Castle, DE, USA) equipped with a rectangular geometry, used for axial tension consisting of two axial clamps that hold the material when the force is applied. Samples were taken from the nanofibrous electrospun matrices and films maintaining a length/width ratio around 2.69 (suggested value in the rheometer procedure). The results are expressed as the Stress σ (Pa) = Force applied (N)/section area (m^2^).

Break tests were performed using the same geometry, starting from a zero-applied force until the material presents a breaking point, with a deformation rate of 0.01 mm/s. The experiments were carried out at constant temperature around 25 °C and a special device was adopted to maintain the relative humidity in the sample environment.

The rheometer also allowed obtaining of the thickness of the samples by measuring the gap between the two plates when they approach film or fiber mat as close as possible until the detector perceives a minimal axial force during compression. This measurement was repeated with a micrometer (Mitutoyo Digimatic micrometer; −25 mm with precision of 0.001 mm) giving very close values. Both techniques used to determine the thickness are in good agreement with a precision of 1 μm.

## 3. Results and Discussion

Mixture of oppositely charged polyelectrolytes form a complex stabilized by ionic linkage. This mixture usually forms a coacervate difficult to process with a stability depending on the pH (for weak electrolytes) and external salt concentration. It was important to find conditions for a homogeneous solution able to be processed by electrospinning. Using the same experimental conditions, casted films and electrospun nanofibers were produced. The use of a film as model permits easier characterization and development of the methods adopted to optimize the experimental conditions.

### 3.1. Film Casting and Characterization

**Figure 1:**
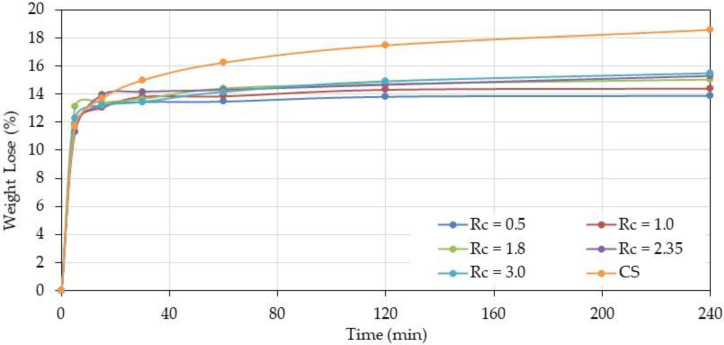
Weight loss of polyelectrolyte complex (PEC) at different chitosan (CS)/hyaluronan (HA) charge ratios *R*_c_ compared with pure chitosan (CS) prepared in water (W)/formic acid (FA) (50/50 *v/v*) as a function of time at 120 °C.

Films with different –NH_2_/–COONa weight and charge ratios are casted on a Teflon surface and dried until constant weight at ambient conditions. The thickness of the dried films is found around 50 μm and strong dependence of the charge ratio is not observed probably due to the similar polymer densities of CS and HA. Chitosan was also treated for comparison with the complexes. The films were submitted to a thermal treatment at 120 °C for 4 h after a study of the dehydration kinetics (Figure 1). Then the degree of swelling in aqueous medium and the eventual solubility are determined before and after thermal treatment.

#### 3.1.1. Kinetic of Thermal Treatment

Over *R*_c_ = 1, the behavior of complexes is similar for the different ratios increasing progressively with the time and chitosan content indicating a larger crosslinkage degree due to H-bonds and probably amide bond formation involving free –NH_2_.

Comparison with chitosan shows that weight loss is higher for free chitosan than for complexes due to lower interaction between chains (thermal treatment of chitosan induces an increase of crystallinity) and lower degree of reaction with residual formic acid used as solvent.

#### 3.1.2. Solubility and Swelling of PEC Films

**Figure 2:**
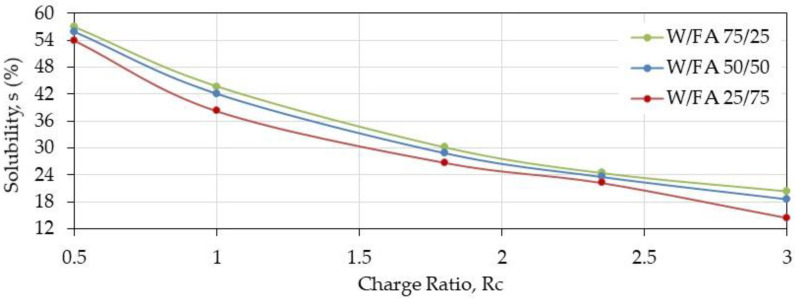
Solubility of PEC films at pH = 7.4 after thermal treatment as a function of charge ratio prepared in different solvents (W/FA 75/25, 50/50, 25/75 *v/v*).

As shown in Figure 2, the three solvent mixtures give nearly the same film characteristics when the degrees of swelling and solubility are considered. Nevertheless, the W/FA 50/50 *v/v* is preferred for the film tests and electrospinning considering that the processing of PEC solutions was difficult in W/FA 75/25 and due to high volatility of W/FA 25/75 which affected significantly the production of fibers.

**Figure 3:**
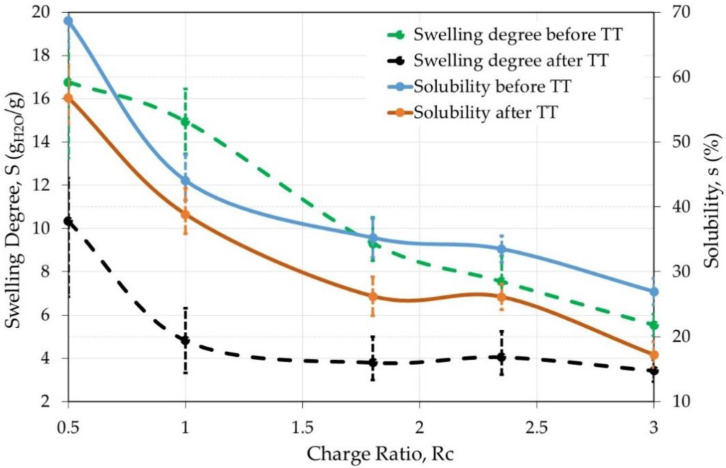
Influence of the thermal treatment (4 h at 120 °C) on PEC films solubility and degree of swelling as a function of charge ratio at pH = 7.4. Average values shown after repeating the experiment 4 times.

Then, the swelling characteristics of the films prepared from complex solubilized in W/FA (50/50 *v/v*) are tested before and after thermal treatment (Figure 3).

The swelling degree decreases strongly after thermal treatment when the charge ratio is lower than 2. At the same time, the solubility also decreases when the charge ratio increases but with a lower efficiency. Thermal treatment clearly allows to stabilize the PEC films which remain easy to handle even in the wet state. In the following, films after a thermal treatment are tested in different conditions of solvent and pH (Table 1).

**Figure 4:**
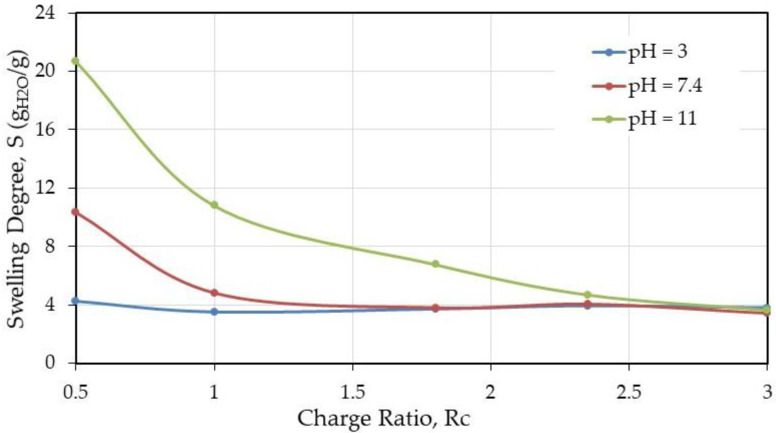
Swelling degree (g water/g dried material) as a function of the charge ratio after thermal treatment.

**Figure 5:**
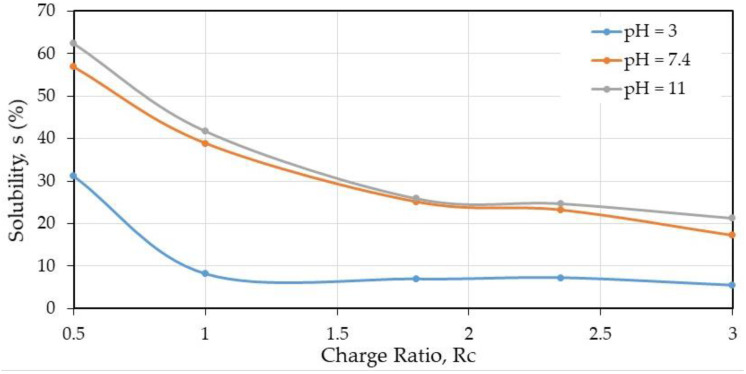
Partial solubility (%) at different pH on PEC films prepared in W/FA 50/50 *v/v* as a function of charge ratio.

From Table 1, few values are presented in the next figures. In Figure 4 and Figure 5, the influence of the charge ratio on swelling degree and solubility are represented, respectively, for different pH after thermal treatment when the solvent used was W/FA 50/50 (*v/v*).

From these results, it is shown that the thermal efficiency starts with a ratio NH_2_/COOH equal to 1.8, indicating the need of enough chitosan in the blend to complex HA, due to the fact that HA is a polysaccharide highly soluble in aqueous medium in a wide range of pH values.

The role of the aqueous medium pH chosen to test the stability of the materials indicates that a general trend is that stability is larger at pH = 3 as soon as the charge ratio NH_2_/COOH is larger than 0.5 independently of the composition. Taking care of the material composition, over *R*_c_ = 1.8, less than 10% is soluble when the material contents 43.3% (*w/w*) of chitosan and consequently 56.6% (*w/w*) of HA. This condition corresponds at lower solubility of HA and gel-like behavior [11] preserving the complex formation.

At pH = 7.4 and 11, the values are close as shown in Figure 5 when chitosan alone becomes insoluble. The solubility is around 22% indicating that the material is stabilized and remains a stabilized polyelectrolyte complex.

The study on films as a model allows, after thermal treatment, to conclude that the complex formation stabilized the material taking benefit of HA as well as chitosan biological properties for new applications.

#### 3.1.3. Film Composition by NMR and Influence of Thermal Treatment

**Figure 6:**
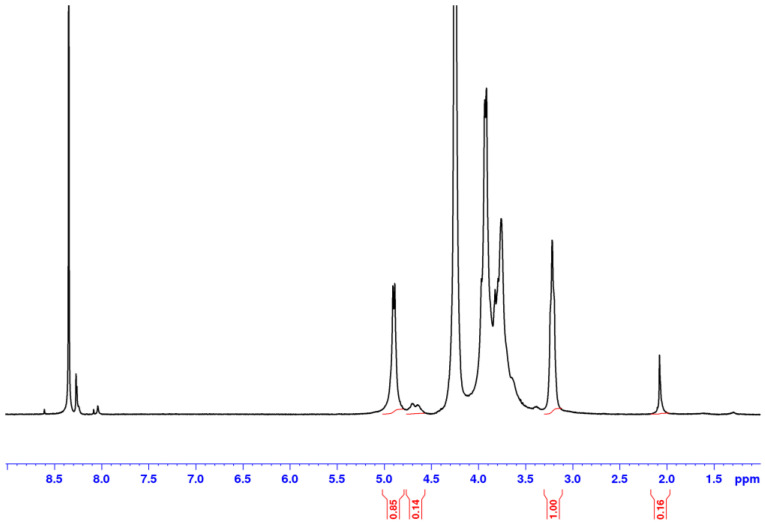
^1^H NMR spectrum obtained on a chitosan film casted from chitosan in D_2_O/DCl solvent at 85 °C.

In a first step, ^1^H NMR of chitosan was examined as reference (Figure 6). The spectrum is obtained after dissolution in acidic D_2_O of a film prepared in formic acid W/FA (50/50).

This spectrum allows to determine the degree of acetylation of the chitosan obtained from the ratio between the signals of the methyl group at 2.1 ppm and H-2 at 3.2 ppm. One gets DA = 0.05. In addition, it shows that there is some formic acid left (around 8.4 ppm) before thermal treatment [34].

After thermal treatment, the film becomes insoluble in acidic medium probably due to H-bond network formation and reinforced by partial crystallization after solvent evaporation as shown by X-rays diffraction (data not shown) [21]. Our data agree with the X-rays spectra given in Figure 2 in Petrova et al. for chitosan/PEO [21]. In addition, as proposed in the literature, amide bonds involving the solvent may occur, decreasing free NH_2_ content and increasing the H-bonds density [28,29,30,31,32].

**Figure 7:**
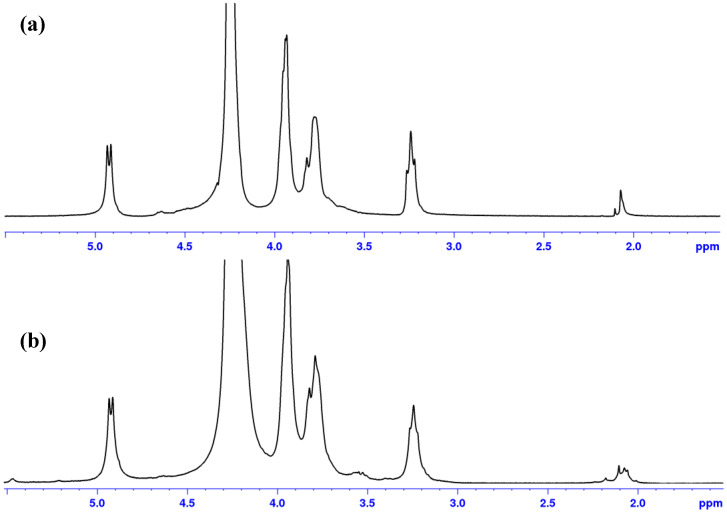
Influence of thermal treatment on ^1^H NMR spectrum for film of chitosan obtained after chitosan solubilization in HCl in absence of thermal treatment (**a**); and after thermal treatment (**b**). Solvent D_2_O/DCl at 85 °C.

It was also demonstrated that the acid used to dissolve chitosan and cast films influences the solubility of films after thermal treatment. A chitosan film dissolved in presence of HCl and dried remains soluble in aqueous medium even after thermal treatment as shown in Figure 7.

After thermal treatment, the films turned brown but remained soluble in acidic conditions even if a small insoluble fraction is formed indicating few strong H-bond interchain interactions. Signal corresponding to –CH_3_ groups around 2 ppm is modified (Figure 7b) and new signals appear around 3.5 ppm with a decrease of the –H_2_ signal at 3.2 ppm indicating probable H-bonds involving the small amount of –NH–CO–CH_3_.

As carboxylic acids (formic acid and acetic acid) formed an insoluble material after thermal treatment, they probably form, as proposed previously, an amide bond with free –NH_2_ chitosan increasing the H-bonds network density as found with peptidic groups [30,31,32,33].

Acid formic solvent was selected due to easier feasibility to produce nanofibers by electrospinning compared to acetic acid used previously [4,5]. Whatever the W/FA ratio (mainly over 25/75), electrospinning allows to process chitosan alone, HA/PEO, as well as the chitosan/hyaluronic acid complex. Then, W/FA was selected as the most convenient solvent for processing our new materials even in absence of other additives such as DMF, N-methylpyrolidone, or NH_4_OH as proposed in literature. Only PEO, extractible in water, was added to favor electrospinning.

#### 3.1.4. Film Made of Complex and Influence of Thermal Treatment

**Figure 8:**
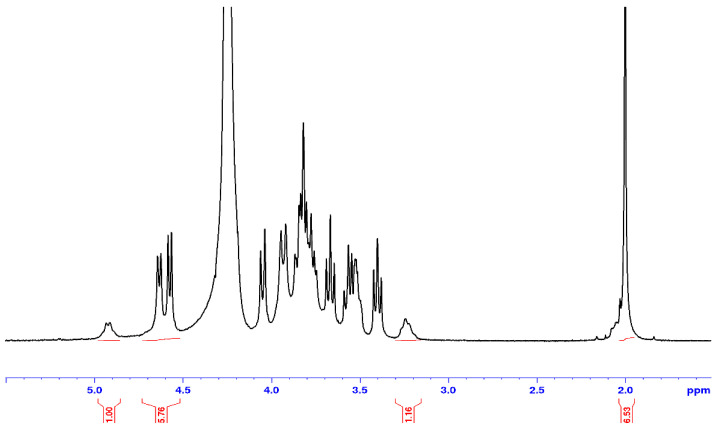
^1^H NMR spectrum of a complex film prepared at a charge ratio *R*_c_ = 0.5 formed in W/FA (50/50 *v/v*) after thermal treatment. Solvent D_2_O/DCl at 85 °C.

In the following, a polyelectrolyte complex prepared at a charge ratio, *R*_c_ = 0.5, was dissolved in D_2_O, DCl after thermal treatment (Figure 8). This sample remaining soluble in acidic conditions can be analyzed as proposed previously [33].

Taking into consideration 2 H-1 of HA at 4.5–5 ppm (one of the units being carboxylated) and the H-1 of chitosan (corresponding to free –NH_2_) at 4.9 ppm, there comes a charge ratio CS/HA = 0.54 in agreement with the stoichiometry of the solution prepared. A large signal occurs around 2 ppm due to –CH_3_ from *N*-acetyl-ᴅ-glucosamine unit of HA (superposed with the small –CH_3_ signal from chitosan).

To conclude, in large excess of HA, the film is still soluble in water or acidic conditions even after thermal treatment. After thermal treatment at higher charge ratio, the films are more stable and only a small amount is solubilized in acidic conditions. It comes that the liquid NMR is only valid in some specific conditions to characterize the composition of the new materials prepared due to the insolubility in aqueous medium when complex forms.

### 3.2. Film Mechanical Properties

#### Influence on Thermal Treatments

Mechanical behavior of complexed and chitosan films was determined at ambient temperature before and after thermal treatment. In each case, the thickness of the material is given for the experimental curves.

**Figure 9:**
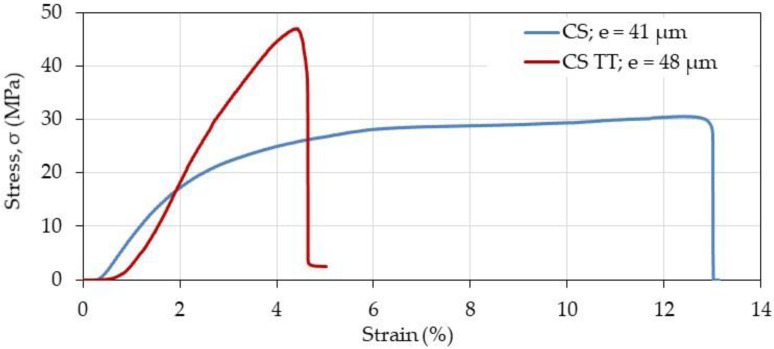
Effect of thermal treatment (TT) on mechanical chitosan film response in the dried state. The thickness (e) of the film samples was found around 40–50 μm.

**Figure 10:**
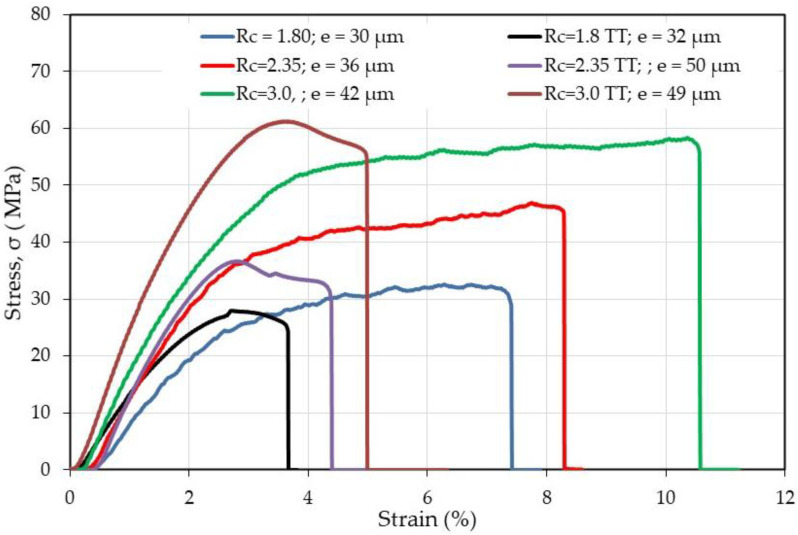
Effect of thermal treatment (TT) on mechanical response of PEC films in the dried state. Stress as a function of strain (%) before and after thermal treatment for different complexes characterized by *R*_c_.

From Figure 9 and Figure 10, it is shown that thermal treatment on CS alone and complexed films increases the stiffness and decreases strongly the plasticity of the materials. In addition, the presence of HA increases the modulus of the films compared with CS alone especially before thermal treatment when *R*_c_ > 1.8. The higher the *R*_c_, the better the mechanical properties.

**Figure 11:**
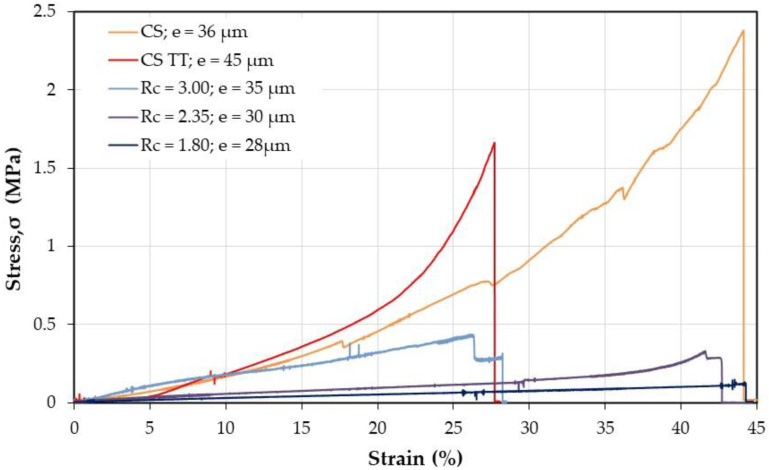
Effect of thermal treatment on mechanical response of chitosan and PEC films in the wet state after stabilization in PBS at pH = 7.4.

Then, it was interesting to also investigate the material performances in the wet state. Some experimental data are given in Figure 11.

For determination of the mechanical properties on films after thermal treatment, the complexes and the treated CS were directly stabilized in PBS buffer but for CS before thermal treatment, it was necessary to firstly neutralized CS in alkaline medium before PBS immersion as suggested beforehand [4,5]. From data in Figure 11, it is concluded that the material is able to be manipulated only when *R*_c_ ≥ 1.8 corresponding to lower values of swelling and solubility. When the charge ratio *R*_c_ increases, the stiffness increases (modulus increases and breaking strain decreases) remaining in all cases slightly lower than for CS alone for samples with the same range of thickness.

These data confirm that, for the first time to our knowledge, stable new biomaterials based on PEC involving HA and CS are obtained in PBS at pH = 7.4, for biological applications.

### 3.3. Nanofibers Production

Low spinnability is observed in biopolymers which are frequently blended with another polymer such as PLA, PCL or PEO. In this work, the former, being widely reported in the literature, was mixed with the prepared PEC solutions allowing nanofiber formation by electrospinning of the CS–HA blend in different charge ratios. As long as the proportion of PEO in the final polymeric mixture was equal or larger than 20% (*w/w*), fibers were obtained; other characteristics like uniformity (beadless morphology) increased with the chitosan content in the initial PEC solution. In our knowledge, it is the first time that nanofibers are produced with the CS/HA polyelectrolyte complex with variable controlled charge ratio.

#### 3.3.1. Optimal Conditions

In order to optimize the production of nanofibers at high yield in PEC, different experimental conditions were explored. In Table 2, the average of the more appropriated parameters for the electrospinning process to get fibers in absence of spray or beads are presented. The fibers are obtained on solution in formic acid /water 50/50 *v/v* for chitosan and chitosan/hyaluronic complexes.

**Figure 12:**
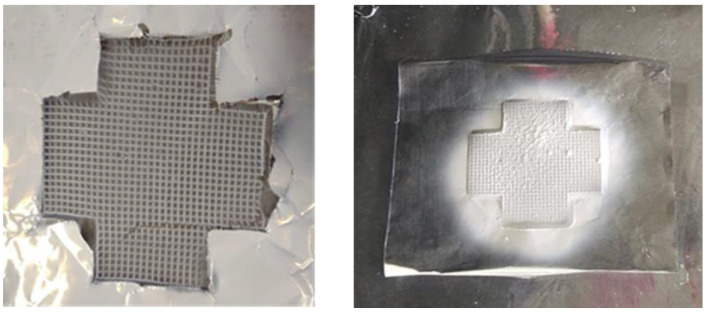
CS/HA+ polyethylene oxide (PEO) nanofibers on metallic collectors utilized for the electrospinning process.

For this electrospinning process, the collector was designed permitting to recover the fibers matrix from a metal plate avoiding sticking on the support. Aluminum foils cut in cross have been chosen as support to remove the mat after processing as shown in Figure 12. In these conditions, the probes for mechanical tests are easy to take out.

Proton NMR of the nanofibers obtained after solubilization for some charge ratios in acidic D_2_O to get the composition was realized for polyelectrolyte complex Chitosan/HA/PEO blends [4]. The spectra are analyzed as done for films obtained in the same conditions to determine the composition of the soluble fraction but also the yield in PEO extractible such as to follow purification of the fiber mats. The essential difference between film and fibers is the presence of PEO signal at 3.7 ppm in fiber samples.

It is usually observed that solubility of the material increases in presence of HA compared with chitosan alone. After thermal treatment, the fraction of insoluble material increases.

#### 3.3.2. Stability of CS/HA/PEO Nanofibers in Aqueous Medium. Effect of the Thermal Treatment on the Solubility and Water Retention.

As spun PEC/PEO 70/30 nanofiber sample with *R*_c_ = 2.35 and 3.0 are treated at 120 °C for 4 h. The weight loss is given in Table 3 as control and being able to be compared with results obtained on films. Then, after several rinsings with ethanol/water 80/20 *v/v* to extract PEO, the samples were dried at room temperature and immerged in PBS at pH = 7.4 to calculate the solubility and swelling degrees under these conditions (Table 3).

From these data, it comes that the weight loss due to thermal treatment is around 10% slightly lower than 14% found on films as soon as the charge ratio *R*_c_ ≥ 2.35. This difference may be attributed to presence of PEO decreasing the interchain crosslinkage available between CS and CS/HA by amide bonds.

It is also shown that PEO corresponding to 30% of the polymers in the blend is extracted by treatment with ethanol/water mixture (80/20 *v/v* in which both HA and CS are insoluble) with some lack of precision due to the small quantity of sample tested each time (around 3 mg dried nanofibers) preserving the complex.

Compared with the data obtained on films, at pH = 7.4, the swelling is slightly lower (it was obtained 4%) and the solubility decreases (it was found 22%). This result may be due to a lower contact time in the aqueous medium or to a favored electrostatic interaction between HA and CS when PEO is extracted. This mechanism is confirmed by larger stiffness of the fibers when PEO is extracted.

#### 3.3.3. Fiber Diameter Determination

**Figure 13:**
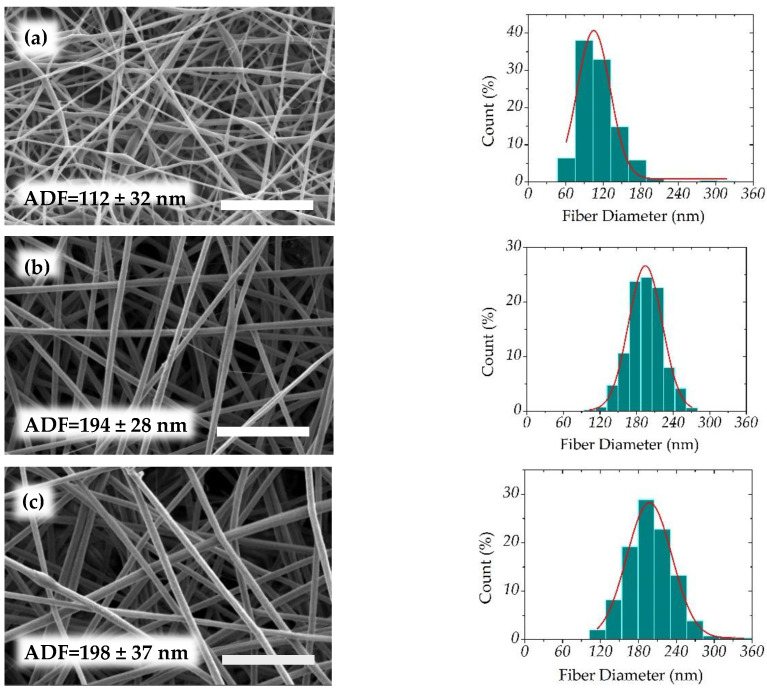

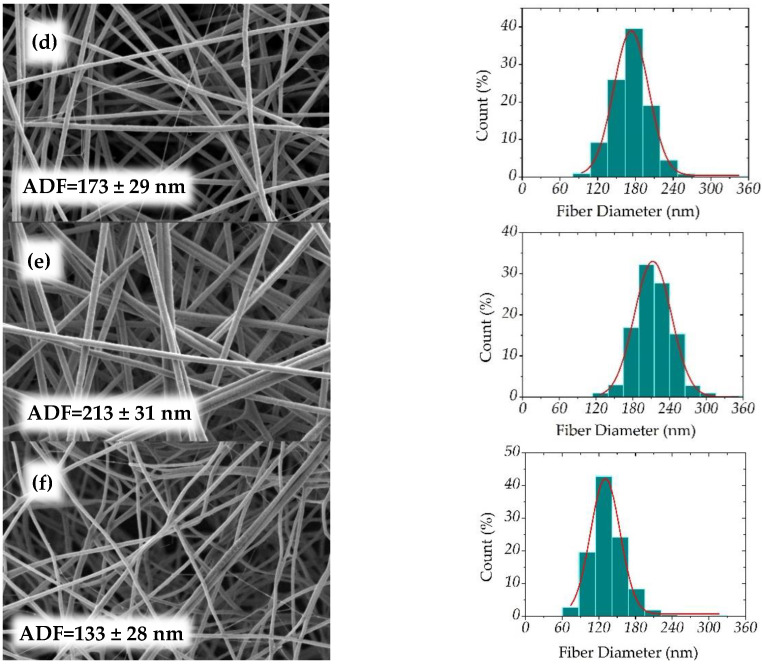
Scanning electron microscopy images of nanofibers obtained at different charge ratios and corresponding average fiber diameters (AFD) (nm) together with their diameter distribution. (**a**) *R*_c_ = 0.5, (**b**) *R*_c_ = 1.0, (**c**) *R*_c_ = 1.8, **(d**) *R*_c_ = 2.35, (**e**) *R*_c_ = 3.0, and (**f**) CS. Scale bar = 3 μm.

The polyelectrolyte complex solutions in W/FA = 50/50 *v/v* are electrospun in optimal conditions previously described. The fiber diameter distributions were calculated and shown in Figure 13.

To show the influence of the PEC composition on the fiber characteristics, the average diameters (ADF) are determined in Figure 13, and compared with produced CS/PEO fibers. It is found that, at higher content of HA (*R*_c_ = 0.5), fibers are thinner in relation with a lower viscosity of the blend at constant total polymer concentration. Then, the diameter increases when the chitosan yield increases and viscosity increases due to polyelectrolyte loose interaction. The average diameter becomes larger than for pure CS in the same experimental conditions. This behavior pointed out the interest of the solvent selected avoiding phase separation between the two oppositely charged polymers. All average diameters for complex nanofibers are close to 200 nm.

#### 3.3.4. Mechanical Properties

Traction experiments were performed on samples having nearly the same thickness in the absence of thermal treatment, but it must be considered that the density of the nanofiber mat is much lower than for the films as discussed previously [5].

**Figure 14:**
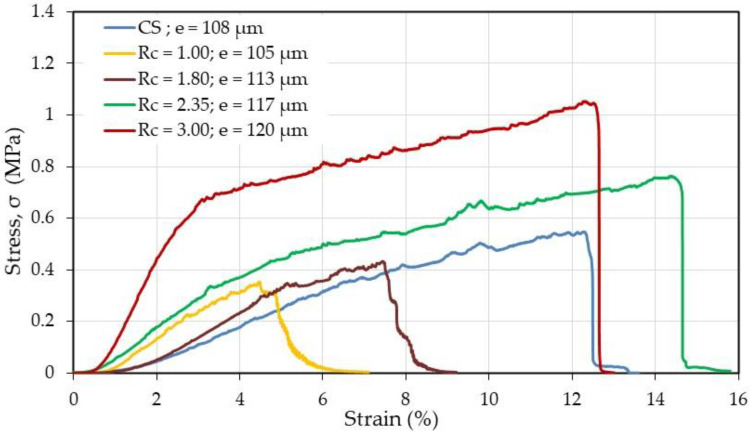
Mechanical behavior of chitosan and as-spun PEC fiber samples in dried state.

Due to lower density of the mat compared with dense films, the values obtained for the mat look low. Firstly, it is shown that the PEC fibers are stronger than CS fibers as soon as *R*_c_ is higher than 1.8 with a relatively large strain at break probably connected with the presence of PEO. The stress at break is increasing directly as a function of the chitosan content. Considering the geometric characteristics of the samples (surface, weight, and thickness of the probe), the density of the mats is compared with that of the film (it is related with the active transverse section of the probe tested). From the cross section ratio estimated from the material density and found around 30 (Table 4), the mechanical performance of nanofibers is in relatively good agreement with that of films: for *R*_c_ = 3, stress at break is 1 MPa and 12% strain (Figure 14) but on compact film it is around 60 MPa and 10% (Figure 10), respectively.

The large porosity and low density of fibers in the mats considerably decrease the effective stress of the mat which may be used thicker if needed.

## 4. Conclusions

In this work, for the first time, we succeeded in processing new biomaterials based on homogeneous PEC blends of chitosan and hyaluronan under casted films and electrospun nanofibers. Hybrid materials made of chitosan coated with HA were also produced. PECs are considered as interesting precursors for materials with biological applications due to the specific characters of the two macromolecules involved.

The solvent adopted is a mixture of formic acid and water, the best composition proposed for electrospinning in the presence of PEO is the mixture 50/50 *v/v*. The influence of the –NH_2_/–COONa molar ratio (directly related with the total potential charge ratio) was examined playing an effective role on the physical properties of films and fibers: solubility and degree of swelling in aqueous medium at different pH, mechanical performances in traction and breaking stress in dried and swollen states. For characterization purposes, casted films were prepared as a model to establish the main parameters on the PEC properties.

The application of the thermal treatment, at 120 °C for 4 h, confirms that it stabilizes the material by decreasing the aqueous medium solubility and swelling degree and increasing the mechanical performances. This effect is founded on the hypothesis of amide and H-bonds formation involving –NH_2_ and –COOH functions. Over a charge ratio larger than 1.8, the swelling and solubility are lower after thermal treatment with 22% and 4%, respectively at pH > 7. The values are lower in acidic conditions where HA forms an H-bond network [11]. Considering its degree of solubility and the mass ratio for *R*_c_ = 1.8 equals 0.77, it is concluded that the insoluble material fraction consists of the HA/CS complex, allowing to take benefit of the specific properties of the two important biological polymers, HA and CS.

These results in relation with the characterization of the new materials prepared in this study could allow the use of materials based on CS–HA PEC at physiological conditions towards biomedical applications such as more efficient tissue engineering scaffolds and drug delivery systems. Additionally, as shown before, the nanofibrous structure promoted the attachment of human osteoblasts and chondrocytes and maintained characteristic cell morphology and viability when dense film is compared with nanofibrous structure [35]. Then, this nanofibrous matrix with low fiber diameters (around 200 nm) and large porosity (density around 0.04 g/cm^3^) is of particular interest in tissue engineering for controlled drug release and tissue remodeling.

To conclude, further work with such nanofibers could be surely envisaged due to fiber mat unique properties like large surface area and high porosity, as well as the enhancing in adherence, growth, and proliferation of seeded cells. In this way, CA-HA nanofibers could help to the successful development of tissue-engineered supports that could also control chondrogenesis in vitro and in vivo (important in cartilage regeneration) [21]. It was demonstrated a significantly higher number of living cells on the surface of the CS–HA hybrid biomaterials, proposed as artificial 3-D ECMs, showing a better biocompatibility compared with CS [21,25].

## Figures and Tables

**Table 1:** Influence of Charge Ratio *R*_c_ and pH on Swelling Degree and Solubility (%) before and after Thermal Treatment for Solubilization in W/FA 50/50.

Charge Ratio (*R*_c_) NH_2_/COOH	Weight Ratio NH_2_/COOH	pH	Swelling Degree (g Water/g) before Thermal Treatment	Solubility (%) before Thermal Treatment	Swelling Degree (g Water/g) after Thermal Treatment	Solubility (%) after Thermal Treatment
0.5	0.21	3	3.2	24.1	4.6	37.2
		7.4	16.8	68.7	10.0	59.3
		11	----	High *	22.6 *	69.5 *
1	0.42	3	2.6	10.1	3.6	11.5
		7.4	14.9	44.0	4.7	37.7
		11	21.8	61.0	11.4	39.4
1.8	0.77	3	4.5	14.5	3.8	9.8
		7.4	9.3	35.2	3.9	25.1
		11	16.0	45.1	6.8	25.9
2.35	1.0	3	6.6	37.1	3.8	8.4
		7.4	7.6	33.5	4.3	23.2
		11	13.9	35.4	4.3	21.3
3	1.26	3	7.0	46.9	3.4	7.1
		7.4	5.5	26.9	3.6	17.2
		11	12.1	28.5	4.1	21.3

(*) Approximative values due to difficult sample handling.

**Table 2:** Experimental Conditions for Electrospinning.

Charge Ratio NH_2_/COOH	Weight Ratio NH_2_/COOH	Pump Rate (mL/h)	Tip to Collector Distance (cm)	Applied Voltage (kV)	Electrospun Products
0.5	0.21	0.08–0.12	17	18–25	Fibers, few beads
1.0	0.42	0.15–0.2	16–17	24–26	Fibers
1.8	0.77	0.15–0.2	17	24	Fibers
2.35	1.0	0.10–0.15	16–17	21–29	Fibers
3.0	1.26	0.12–0.17	16–17	21–23	Fibers

**Table 3:** Weight Loss for Thermal Treatment at 120 °C for 4 h and Stability of PEC/PEO Nanofibers for *R*_c_ = 2.35 and 3.

*R*_c_	Weight Loss (%) after TT	Remaining Polymer (%) after EtOH/H_2_O Washing	Swelling Degree (gH_2_O/g) at pH = 7.4	Solubility (%) at pH = 7.4
2.35	9.8 ± 2.5	69.8 ± 8.1	3.3 ± 0.3	12.2
3.0	10.9 ± 0.4	73.79 ± 0.18	3.7 ± 0.5	13.9

**Table 4:** Morphological Characteristics of Films and Fibers Tested in Mechanical Experiments. Influence of the Charge ratio, *R*_c_.

Composition	Average Density (g/cm^3^)		Ratio Density Film/Fibers
	Casted Film	Electrospun Nanofiber Mat	
CS	0.846	0.0295	28.7
*R*_c_ = 1.0	------	0.0383	----
*R*_c_ = 1.8	0.949	0.0284	33.4
*R*_c_ = 2.35	1.127	0.0395	28.5
*R*_c_ = 3.0	1.163	0.0512	22.7
